# A Trauma Doctor’s Practice in Nineteenth-century China: The Medical Cases of Hu Tingguang

**DOI:** 10.1093/shm/hkw075

**Published:** 2017-05-01

**Authors:** Yi-Li Wu

**Keywords:** China, Qing dynasty, bone setting, surgery, traumatology

## Abstract

This paper analyses the medical activities of Hu Tingguang, an early nineteenth-century Chinese healer who specialized in treating traumatic injuries. Hu aimed to improve the state of medical knowledge about injuries by writing a comprehensive treatise titled *Compilation of Teachings on Traumatology*, completed in 1815. This work notably included a set of medical cases describing the experiences of Hu and his father, which Hu used to teach readers how to employ and adapt different therapies: bone setting, petty surgery, and drugs. By examining how Hu dealt with different forms of damage to the body’s material form, this paper shows how manual therapies could be a focus of medical creativity and innovation. It also contributes to a growing corpus of scholarship exploring the way that awareness of and concern with the structure of the body historically shaped Chinese medical thought and practice.

## Introduction

It began as a playful challenge between friends: who was the stronger? But as the two men were gripping hands and trying to force each other backwards, one man’s upper arm bone suddenly snapped with a crack. ‘The injured person was in so much pain, he thought he would die,’ recalled Hu Tingguang, the doctor they called on to help. But Hu assured them that there was ‘nothing to fear’. Hu manually pressed the broken back into alignment, ‘smooth and proper’, and applied a medicinal plaster to the site of the fracture. Then he took strips of bamboo, wrapped them in paper, and placed them around the arm as splints. Finally, he wound a cloth wrapper around the outside of the bamboo strips to fix them firm and tight. After a month, Hu reported, the injured man’s arm was fully healed, and so was the friendship.

Hu Tingguang recorded this case of the arm-wrestling friends in his book, *A Compilation of Teachings on Traumatology* (*Shangke hui zuan*), completed in 1815 (hereinafter ‘*Compilation*’).^1^ It was one of a set of cases recounting the experiences of Hu and his father, both specialists in the treatment of traumatic injuries. Although the Chinese medical landscape was historically well-peopled with practitioners like the Hus, few such healers prior to the twentieth century left writings that revealed not only what they did in practice, but also how they thought about what they did. Hu Tingguang’s text is thus valuable for shedding light on an aspect of Chinese medical history that has been relatively understudied: how healers treated damage to the structural integrity of the body. Existing histories of Chinese medicine have examined infectious diseases and functional disorders (epidemics, plague, beriberi, leprosy, smallpox, madness)^2^ as well as ailments afflicting specific body parts and populations (skin diseases, eye diseases and gynecological and pediatric maladies).^3^ In the People’s Republic of China today, so-called ‘osteology and traumatology’ (*gu shang ke*) is also a standard clinical division in schools and hospitals of ‘traditional Chinese medicine’ (TCM), namely the modern-day iteration of historical practices. But while Chinese practitioner-authors have amply documented the richness of this healing legacy, we still lack a critical and historically-sensitive account of its development.^4^ A study of ‘traumatology’ (*shangke*), Hu Tingguang’s claimed area of specialisation, thus allows us to consider an important realm of bodily suffering and its place in Chinese medicine.

More broadly, a study of the injured body allows us to reconsider the significance of body structure in the historical development of Chinese medical thought. Today, TCM is a form of alternate and complementary medicine, primarily focusing on chronic dysfunctions that biomedicine cannot cure.^5^ As scholars have shown, however, this particular configuration arose from attempts by twentieth-century Chinese doctors and policy makers to preserve and promote indigenous medical ideas in a world dominated by biomedicine.^6^ A key challenge was to justify the seemingly irreconcilable differences between Chinese medical descriptions of the body and those of Western anatomical science. The explanation that developed, which still dominates present-day views, is that Chinese medicine was not actually interested in bodily structure, but rather in the transformations and functions of qi, the material force that underlies and enables all cosmic phenomena.^7^ However, as I have recently argued, Chinese medical thinkers historically did, in fact, investigate body structure, even if they did not practise dissection.^8^ Furthermore, the idea that Chinese medicine is primarily for chronic conditions should be understood as a product of the growth of state medicine after the establishment of the People’s Republic of China.^9^ Historically, Chinese doctors routinely treated acute and life-threatening conditions. But the growth of biomedicine-dominated hospitals marginalised Chinese medicine in the treatment of emergencies. To survive as a profession, Chinese doctors were compelled to focus instead on chronic illnesses.

Hu Tingguang’s *Compilation* takes us back to a time when Chinese healers expected to treat acute conditions, including injuries. To heal injuries, the doctor required not just knowledge of qi transformation, but also knowledge of body structure and how to restore it. This was particularly evident in the Hu family’s forte, bone setting. This paper will thus use Hu’s text as a case study of the ways in which doctors in late imperial China engaged with the material and structural aspects of the human body. Not only was Hu concerned about damage to bodily structures, but his *Compilation* shows that this structural body could become the focus of therapeutic and epistemological innovation. I will begin by describing the history of the Hu family and Hu Tingguang’s text. Next, I will survey the Hu family medical cases and the way Hu selected from different manual and pharmacological techniques. Finally, I will focus on Hu as a bone setter, with particular attention to his treatment of shoulder and hip dislocations. A special problem was how to treat female patients, given that norms of gender propriety nominally forbade the male doctor to touch the female body.

## Hu Tingguang and his *Compilation*

Hu Tingguang was a native of Xiaoshan County in Shaoxing Prefecture, located in the region of Jiangnan (‘south of the Yangzi River’), the cultural and economic heartland of late imperial China. In his *Compilation*, Hu says that his main occupation as a youth was ‘studying books’, namely pursuing a classical Confucian education to prepare for the civil service examinations that qualified men for government positions and thus entry into the ranks of imperial China’s socio-political elite.^10^ The fact that Hu ended up practising medicine indicates that he was one of the many men of his time who took up medicine when they failed to advance in the examinations, a choice that in his case would have been facilitated by his family background.^11^ Hu Tingguang’s father was a doctor, and presumably Hu’s grandfather as well, because Hu notes that he is the third generation in his family to practise traumatology.^12^ Historically, there were many such families of ‘hereditary doctors’ (*shi yi*) for whom medical expertise was a kind of economic patrimony handed down through the generations. At the same time, those who had the means would pursue a classical education for their sons. Hu Tingguang’s father was also literate, instructing Hu to read certain books, and personally hand copying medical works that he found useful.^13^

In late imperial China, the practice of medicine was regarded as a technical art, and it did not in itself confer much social or cultural prestige.^14^ Literate doctors seeking to elevate their status thus emulated the intellectual and cultural ideals of the Confucian scholar, emphasising classical studies, mental perspicacity and moral cultivation. Not surprisingly, they assigned the greatest prestige to healers who worked with their minds, namely those who drew on their knowledge of cosmological dynamics to compose prescriptions of drugs tailored to each individual patient. As experts in manual healing, the Hu family would have ranked low on this imagined hierarchy of doctors, but as literate men, they would have enjoyed higher social status relative to other manual healers. In terms of social class, their clients ranged from peasants and labourers to members of the scholarly elite.^15^ Their access to upper class patients was likely facilitated by a combination of literati identity and native place. Hu’s home prefecture of Shaoxing was famous for producing *muyou*, namely government secretaries and experts in legal and fiscal affairs.^16^ Hu Tingguang spent some time in the imperial capital of Beijing, and was in fact staying there when he wrote the preface to his *Compilation* in 1815*.* Hu also notes that he was on close terms with a group of men from his hometown who were working at the Ministry of War. Thanks to these connections, Hu was asked to treat the wife of a ministry clerk when she broke her leg.^17^ Hu’s ability to secure two laudatory prefaces for his *Compilation* from scholar-officials in the capital also suggests a good network of literati connections.^18^

### Composing the *Compilation*

Hu’s dual identity as a literatus and hereditary doctor shaped his approach to writing the *Compilation*. Comments that Hu made in his preface and ‘guide for the reader’ (*fanli*) show that he intended his work to be a landmark intellectual achievement, one that would synthesise and expand the state of knowledge about the treatment of injuries. In his preface, Hu noted that there was a dearth of specialised texts on the subject. While his own family possessed a useful text that he referred to as ‘Master Chen’s book of joining bones’ (*Chen Shi jie gu shu*), Hu felt it was too limited in scope.^19^ He therefore undertook to create a comprehensive work on the treatment of injuries, and he spent seven years composing his *Compilation*, writing three successive drafts. Hu’s preface highlighted the voluminous content of his text, which ‘lists 44 classes of injuries, appends over a thousand medicinal simples (*dan fang*), is divided into six classificatory categories (*lei*), and comprises twelve *juan* [book sections]’. Although Hu said that he was paying the woodcarvers to make the printing blocks, it appears that *Compilation* never circulated as a printed work. The only known extant versions are two handwritten manuscript copies, held by university libraries in Beijing and Guangzhou.^20^ Although there are some significant differences between these copies, both bear the inscription ‘edited in the Hall of Bestowing Universal Benefit’ (*Bo shi tang*), the literary name Hu gave to his home, showing that they are derived from a common source close to Hu.^21^ Both also include a printed title page. This suggests that Hu may have indeed started to print the work, but was unable to complete the project.

One of Hu’s goals was to promote the idea that the treatment of injuries constituted a distinct division of medicine, with its own organisational logic. Chinese literate medicine had long recognised different sub-fields or ‘curricula’ (*ke*), each with specialised literature, including ‘the curriculum of women’ (*fuke*, gynecology and childbirth) and ‘the curriculum of children’ (*erke*, pediatric medicine).^22^ The treatment of wounds was historically an important part of medicine as well, discussed in medical works under rubrics such as ‘injuries from falling and beating’ (*die pu sun shang*), ‘wounds from metal [blades] and arrows’ (*jin zu*), and ‘breaking and snapping injuries’ (*zhe shang*). These ranged over topics such as how to wash, dress and bandage wounds; how to set simple and compound fractures; how to anaesthetise patients before carrying out painful procedures; when to needle and when to cut through flesh; how to staunch bleeding and suture injured body parts; and how to promote healing and treat the sequelae of injuries, including what biomedicine would identify as shock and infection. However, Chinese medical texts historically subsumed these topics into a broader category of ailments afflicting skin and flesh, known variously as ‘lesion medicine’ (*yang yi*) or ‘the curriculum of external medicine’ (*waike*), a category that also included ulcers, abscesses, goitres, rashes and tumours.

Hu Tingguang noted that the categorisation of injuries under ‘lesion medicine’ dated back to antiquity, and he sought to separate it out. His compilation strategy was to weave together famous writings on the treatment of injuries, supplementing them with lesser known works as well as his own therapeutic experiences. Hu explained that his ‘warp threads’ (*jing*) would be the bone setting chapters from the *Imperially-Compiled Golden Mirror of the Medical Lineage* (*Yu zuan yi zong jin jian*), compiled by the Imperial Medical Academy (*tai yi yuan*) and completed in 1742.^23^ The *Golden Mirror*’s compilers meant it to serve as a standard of correct practice and it was used to instruct and evaluate doctors at court.^24^ Its last four *juan* were collectively titled the ‘Fundamental Meaning of the Essential Teachings on Rectifying Bones’ (*Zheng gu xin fa yao zhi*). These represented an unprecedented attempt to elevate the treatment of bone-related injuries into a distinct curriculum within literate medicine.^25^ To supplement the *Golden Mirror*, Hu drew his ‘weft threads’ (*wei*) from the writings of ‘all the myriad experts’. An important reference was Xue Ji (1487–1559), a former imperial physician, influential doctrinal innovator, and prolific medical author. Xue Ji’s writings included *Categorised Essentials of Rectifying the Body* (*Zheng ti lei yao*), completed no later than 1548. Later authors routinely described this work as the earliest specialised treatise on injuries. Xue’s text, which included 84 cases drawn from his own practice, primarily focused on what modern doctors would call blunt trauma and soft tissue injuries, particularly injuries from beating and falling. Xue did not set bones, but would treat patients for complications from fractures.

The great breadth of Hu Tingguang’s reading is evident in the range and diversity of the ‘experts’ that he cited in his text. Besides the works of famous Chinese doctors like Xue Ji, Hu cited teachings from the martial monks of the Shaolin Monastery, treatises by palace doctors of Choseon Korea, and a description of the body written by the Jesuit missionary Adam Schall von Bell (1592–1666), who had served as Director of the Imperial Observatory under Emperor Shunzhi (r. 1638–61).^26^ Hu’s book also incorporated illustrations and textual descriptions from the Qing government’s official inquest manual, titled *Records on the Washing Away of Wrongs* (*Xi yuan lu*), initially promulgated around 1741.^27^ This was a greatly expanded version of the famous forensic treatise written in 1237 by judicial official Song Ci, and its compilation represented the first time a Chinese government had established empire-wide official inquest standards. Besides investigating corpses and skeletal remains, Chinese inquests also assessed the wounds of those who had been assaulted and might be in danger of dying. The official manual thus contained detailed descriptions of wounds and bones that Hu integrated into his *Compilation*.^28^

In both the body and title of his work, Hu also deliberately employed a new term to describe the knowledge that he had now synthesized. He noted that earlier experts who wrote on injuries had used the terms of ‘rectifying bones’ or ‘rectifying the body’, clear references to the *Golden Mirror* and Xue Ji. However, Hu felt that his project required a different name: ‘Today, I organise it into a separate curriculum, assembling together things that arise from damage and injury. Therefore, I call this the “curriculum of injuries” (*shangke*).’^29^ To the best of my knowledge, the Chinese term ‘curriculum of injuries’ (what I am translating as ‘traumatology’) was not used in medical literature prior to the early nineteenth century. Besides being used by Hu Tingguang, the term also appeared in the work of Qian Xiuchang, a literate specialist in trauma medicine from Shanghai. Qian completed a text called *Supplementing the Essentials of Traumatology* (*Shangke bu yao*) in 1808. Although much shorter than Hu’s text, it likewise claimed to be an expansion on the *Golden Mirror*, and it also incorporated forensic materials.^30^ Although we cannot definitively say who coined the term *shangke*, it clearly had great significance for Hu Tingguang. Terms such as ‘rectifying the body’ or ‘rectifying bones’ focused on what the practitioner did, foregrounding the manual techniques that he used. By contrast, ‘curriculum of injuries’ highlighted aetiology, a more scholarly approach that embodied a central value of Chinese literate medicine—seeking out and treating the true root causes of illnesses.^31^ Thus, whereas earlier writers might segregate discussions of damaged bones from discussions of damaged skin and flesh, Hu now gathered all these conditions together under the rubric of disorders caused by injury.

### Overview of the *Compilation*

Hu laid out his medical curriculum in 12 numbered *juan* (book sections) plus two unnumbered sections. The *Compilation* emphasised both image and text, manual healing and pharmacology, doctrine and practice. The opening (unnumbered) section presents diagrams of the body and bones (adapted from medical and forensic sources) and illustrations showing how to reduce dislocated bones (Hu’s own creation). These serve as visual references for the work as a whole and correspond to later textual discussions. The next two *juan*, numbered 1 and 2, establish the scholarly pedigree of traumatology by presenting learned medical descriptions of the injured body and its treatment, as well as medical and forensic descriptions of the bones. The next four *juan*, numbered 3 through 6, comprise an annotated commentary on the *Golden Mirror*’s ‘Rectifying Bones’. Hu first presents the *Golden Mirror*’s discussion of manual manipulations and orthopaedic ‘implements and tools’ (*qi ju*), adding many pages of mnemonic verses to help readers remember bone setting techniques. Next, he presents pharmacological remedies (oral and topical) for treating soft tissue injury and the sequelae of injuries, augmenting these with medical cases from the writings of Xue Ji and other learned doctors. Hu then turns to the *Golden Mirror*’s chapters describing the bones and bony landmarks found in each section (*bu*) of the body, as well as how to treat their most typical injuries. Starting with the head, face and neck, the discussion then moves through the chest, back, flanks, arms and legs. Here Hu also compares information from forensic works with descriptions in the *Golden Mirror*. He also adds information about important forms of soft tissue injury, including an entry on cut throats and a discussion of eviscerating injuries.^32^ At the end of *juan* 6, Hu appends his family medical cases.

The remainder of the *Compilation* is devoted to drugs and formulas. *Juan* 7 and 8 comprise a catalogue of prescriptions, arranged according to the form of the medicine and the number of Chinese characters in its name (for example, ‘three character powders’ and ‘four character decoctions’). This is followed by an unnumbered *juan* that presents Hu’s research into Li Shizhen’s masterwork of pharmacology and natural history, the *Systematic Compendium of Materia Medica* (*Ben cao gang mu*, pref. dated 1590). Hu collated the descriptions of individual drugs that Li identified as useful for treating injury, and he arranged them according to the type of injury that they were said to treat. Finally, *juan* 9 to 12 present a catalogue of formulas organised by type of injury. Here Hu also incorporates forensic descriptions of different kinds of wounds and how to distinguish between them.^33^

### The learned bone setter

One of Hu’s aims in constructing a more intellectually sophisticated traumatology was to promote pharmacological knowledge among healers of injury. This was necessary, he suggested, because existing practitioners relied excessively on proprietary methods developed by their families, with scant knowledge of the properties of drugs or the principles of learned teachers:

[I]n the area of traumatology, those [remedies] bestowed by masters are few, and those transmitted within families are numerous. [Practitioners] merely know about the joints of the bone, and they say that this is something that manual methods can be used to treat. But if you ask them about their drugs, they say, ‘this originated from secret methods.’ They deeply cherish their proprietary [knowledge], and they do not know about the other things. Alas, how can this be considered ethical medicine?^34^

More important, however, Hu aimed to disseminate bone setting knowledge to a literate audience and to convince educated doctors that it was a worthy pursuit. The centrality of bone setting to Hu’s project is apparent in the title page for his work. Above the title, *A Compilation of Teachings on Traumatology*, is the phrase: ‘A new book on making bones as good as new.’ To the right of the title are three pairs of phrases that summarise the text’s contents. These also highlight bone setting, mentioning manual techniques before pharmacological ones:

Annotated diagrams of the bones and joints of the entire body;

Ingenious discourses on the knack of treating all the bones

Secret techniques for joining bones and mounting joints;

Refined teachings on using drugs for the concurrent illness patterns

Methods for splinting, wrapping, and using apparatus and tools;

Formulas by category: pills, powders, ointments, and elixirs

Members of the scholar-official class had long considered medical knowledge to be a valuable part of the gentleman’s repertoire, a ‘humane art’ that allowed him to care for family, friends and people under his jurisdiction. Many studied medicine as an amateur pursuit, and some even compiled their own anthologies of useful lore.^35^ However, Hu noted in his preface, the subject of how to treat injuries was relatively neglected. Using Confucian tropes about cherishing one’s body and promoting the well-being of all people, Hu suggested that studying traumatology was also a worthy act of benevolence:

I humbly consider the fact that the hair and skin of our bodies is received from our parents, and we dare not destroy or injure it. People study medicine, and they possess the skills to benefit people, and the desire to benefit the world. And yet when people suffer pain and distress from the injuries of falling and striking, and they are crying out and running about to find succor, are they actually able to encounter people on a par with the ‘Man of Qin and Yue’ [the legendary ancient doctor Bian Que]?^36^

Other medical writers had noted that scholarly doctors tended to look down on manual healing. Lu Shidao, a scholar-official who wrote a preface to Xue Ji’s work on injuries, suggested that ‘the success of joining and restoring resides in the ingenuity of manual methods, so that the labor of pressing and pulling is generally despised as the work of crude practitioners’.^37^ But speaking as a literate healer of injuries, Hu pointed to a more practical impediment, namely that bone setting knowledge was mostly handed down in medical families and lineages, and thus not accessible to those outside these personal networks. As he noted: ‘Unless one has access to the fount of knowledge secretly transmitted through the generations, one cannot obtain the knack and understand the fundamentals.’^38^ Hu aimed to take this orally transmitted knowledge and put it into a form that could be transmitted and studied via texts. In addition to conveying this information through diagrams, mnemonic verses, and detailed descriptions of bones and how to treat them, Hu would teach his readers by sharing his own experiences as a healer.

## The Hu Family Medical Cases

During the sixteenth century, the medical case collection (*yi’an*) became an important textual genre in China. Individual doctors (or their disciples) authored cases to demonstrate their skills or the superiority of certain strategies, while medical learners and seasoned doctors alike read cases to supplement their own store of therapeutic experience.^39^ Hu Tingguang similarly saw medical cases as important pedagogical resources, presenting his readers with a set of 42 selected cases that he said would supplement his descriptions of manual methods. Almost all these cases involved patients treated by Hu Tingguang (33 cases), by his father (6 cases) or by father and son together (1 case). (The remaining two cases involved a person who died after a fall without treatment, and a local butcher who attempted suicide by slitting his own throat and was treated by a government doctor.) Hu explained that he selected them because they involved ‘ailments that are dangerous or unusual, and treatments that are convenient and quick’.^40^ Whereas heuristic teachings presented an idealised ‘curriculum’, these medical cases revealed the healing encounter in all its messy and unpredictable manifestations. Besides describing different surgical and bone setting techniques, Hu’s cases emphasised the need for therapeutic flexibility when employing manual or pharmacological treatments. Notably, he demonstrated for his readers how he himself would improvise and innovate on the basis of existing knowledge.

### Flexible therapies for varied injuries

Hu’s cases show that people in late imperial China got hurt in numerous ways ranging from the mundane to the melodramatic. A number of Hu’s patients were injured in fights: two brothers got into an argument that ended with one stabbing the other in the head, and one of Hu’s cousins was bitten while trying to break up an altercation. Transportation accidents were common: some of Hu’s patients broke and dislocated their bones after falling off horses and oxen, while others were injured when their vehicles overturned. Certain occupations also carried inherent risks: an awning-maker fell from his scaffolding, a bricklayer slipped from a mossy ledge, a military man fell from his horse during a mounted archery test. Some people got hurt going about their everyday business: Hu’s cousin tripped on a tree branch while coming home from school, while a man fell out of bed in the middle of the night reaching for his chamber pot. China’s judicial system, which employed interrogative torture and corporal punishment, also generated its own set of medical problems. One of Hu’s patients was a man who had been flogged. Qing law required the death penalty for unfaithful wives and their lovers, and two of Hu’s patients were suspected adulterers who had cut off their own penises to protest their innocence.

Almost all of these cases involved some kind of obvious damage to the material integrity of the body’s structures, ranging in severity from skin wounds to slit throats. To handle these varied situations effectively, the doctor needed to master many different techniques, and he also needed to know which methods were most appropriate in each case. For example, a standard treatment for bleeding was to apply powdered drugs onto the wound (analogous to modern medicine’s use of haemostatic powders). But Hu’s opening case showed that this did not always work. His patient, a girl of 12, had sustained a violent blade wound to the crown of her head, which continually bubbled out blood.^41^ Other practitioners had already applied topical powders, without effect. Then Hu remembered two manual methods that he had read about for stopping the bleeding from blade wounds: applying a cauterising heat to the wound, and applying a piece of black felt to the wound. He combined the two by heating up a black felt hat, which he bound tightly onto the girl’s head. The bleeding then stopped.

The bleeding girl was cured by manual methods after drugs failed. The converse dynamic appears in the case of a woman of 60 who dislocated her jaw while yawning.^42^ Hu’s father initially reduced it for her, and when it dislocated again, Hu also put it back up for her. But the woman’s jaw repeatedly dislocated. One possible remedy was to secure the jaw with ‘a strap to catch it and hold it in place’. However, Hu’s father judged that this kind of ‘manual technique’ (*shou fa*) was futile, because the fundamental problem was that the woman was suffering from a depletion of qi and thus lacked the vital force needed to ‘hold and bind the cavities of the body’. He therefore administered medicinal decoctions designed to bolster qi. After four doses, the woman’s jaw was stable and no longer came out.

In the case of an injured stonemason, Hu employed manual techniques and drugs in tandem, displaying both erudition and inventiveness.^43^ The mason ‘was laying stone steps, when his thumb was smashed flat by a stone’. The poor man’s pain was ‘indescribable’. Hu happened to be in the area visiting his relatives. But because he had not brought along any drugs with him, he had to improvise. He ordered the mason’s employer to prepare a topical paste consisting of ground Sichuan peppercorns mixed into heated brown sugar. Once this was ready, Hu began his treatment, starting with physical manipulation of the thumb, followed by application of the paste:

I counseled the employer to hold the stonemason fast in his arms. Without regard for his pain, I quickly took hold of his finger and rubbed it [back into] a round [shape]. Then while the pepper sugar was hot, I spread it thickly onto the finger and then wrapped it, using cloth to tightly bind it. Thereupon, his pain stopped, and it did not form pus and it did not swell. After ten days, he was healed.

The mason’s employer, seemingly surprised at the efficacy of these humble substances, asked Hu whether this concoction came out of the remedy books. Hu answered that it was his own creation, and he rejected the snobbish belief that a drug had to be expensive in order to work well. His explanation echoed the ideals of the Confucian doctor, simultaneously demonstrating his knowledge of medical literature, his knowledge of pharmacology and his sympathy for the humble:

Now when using medicines in the homes of the common people, the first principle is to select that which is cheap, and the second is to select that which is convenient. The nature of pepper is acrid and hot. Acrid can dispel, and hot can promote circulation. The *Classified Outline [of Materia Medica]* says that it ‘opens the pores, clears obstructions from the blood vessels, and can be made into a medicinal paste’. The flavour of sugar is sweet and cold. Sweetness can soothe pain, and cold can eliminate heat. In general, when people are injured, there are none who do not have stagnant and impeded [blood] causing heat and pain. Although this method was invented by me, it addressed the illness signs, and today we obtained its efficacy.

In explaining the rationale behind this remedy, Hu also referred to a wider realm of practical knowledge, not recorded in books. He noted that when the ‘beaters of tinfoil’ in the famous nearby city of Hangzhou injured their thumbs, they also treated themselves with ‘cheap and convenient’ drugs, binding slices of Indian mallow (*qing ma*) onto their thumbs. This was effective because mallow also broke up stagnation and enlivened blood.^44^

### Petty surgery

Hu’s therapeutic repertoire also included petty surgical techniques. It seems, in fact, that healers such as Hu were expected to know how to sew up wounds. This is suggested by a case involving one of Hu’s neighbours, a barrel-maker, who severed the tip of his index finger with an axe. He beseeched Hu to sew his fingertip back on, arguing that it was possible to graft branches to trees, so why not reconnect a finger? And indeed, some remedy books had recorded methods for ‘rejoining’ fingers. But Hu rejected the idea as ridiculous. Humans were not trees, he said, and anyway the injury was too severe: ‘A superficial break can be joined up, but today there is not a single thread that is connected. How could one rejoin it?’^45^ Instead, Hu gave him a topical medicine to halt the bleeding and prevent suppuration, and the finger subsequently healed. Although Hu did not suture in this case, it is worth noting that he and the barrel-maker both assumed that sewing up fingers was an entirely normal thing to do, even if they disagreed about the circumstances under which it would work.

Hu deployed his suturing skills in other cases, which show that surgery could be an arena for displaying a healer’s knowledge and virtuosity. One patient was a young boy who had gone swimming in a shallow pond and cut open his belly on a discarded potsherd. His intestines were protruding from the wound, and other healers had been unable to make them go back in. Hu simmered barley in water to make a starchy concoction, spraying this on the intestines to lubricate them. The boy was lying on a rope bed, and Hu instructed his family members to gently rock the bed back and forth, essentially creating an alternating pressure on the left and right of the boy’s torso that made the intestines gradually retract. Hu then sutured the wound closed with a thread made from the inner root bark of the white mulberry, and dusted the outside with powdered ophicalcite. All of these techniques had previously been recorded in medical texts, and Hu’s broad command of the medical literature allowed him to be successful.^46^

Hu’s wide reading also helped him treat a patient who sustained a serious genital injury during a drunken brawl: ‘His kidney sack [scrotum] was torn in pieces, and his two eggs [testicles] hung down in the crotch of his trousers.’^47^ Hu sewed up the torn scrotum with mulberry thread, and dusted the outside with ophicalcite. Several days later, however, the sutures burst open. Hu explained that this was because the patient had failed to stay calm during recovery and had flown into a rage. There was also some kind of putrefaction at work, because Hu noted that ‘the sutured places were all rotten and there was nothing more that could be sewn up’. What to do? Hu then remembered that a certain ‘Master Jinxi’ (*Jinxi Shi*) treated knife wounds with a plaster made from the web of the ‘wall coin’ spider (*bi qian*), so-called because of its disc-like webs.^48^ With a helper holding up the testicles, Hu applied spider webs ‘layer by layer’ to the man’s scrotum until the wound opening was secure. He then ‘dusted it with drugs to regrow flesh and close up wound openings’ and administered an oral potion to clear away pathogenic wind and to facilitate the discharge of liquids. Finally, he forbade the person to move about or lose his calm. After two months, the patient was healed.

Besides repairing structural damage, Hu adapted known methods to ameliorate the condition of permanently damaged body parts. One patient was a monk who was under suspicion of adultery and cut off his penis to demonstrate his innocence. After the stump healed, ‘the urine passage was closed up and small, only enough to admit a thread, and urine dribbled out with extreme difficulty’.^49^ Hu Tingguang considered the possibility of enlarging the opening with a knife or ‘medicated string’ (*yao xian*), this latter being a standard implement for tying off and removing haemorrhoids and warts.^50^ However, Hu worried that such techniques would cause new wounds. Then he remembered that others had used lead pellets to open up clogged ear canals or to ‘open up the aperture of a woman with obstructed vagina’. He thus took some lead and made it into the shape of a needle, and threaded it into the monk’s urethra. ‘Before a ten-day period had elapsed,’ Hu reported, ‘it had enlarged and was unobstructed.’

Minor amputations were also used when an injury could not be healed. Chinese doctors historically recognised the need to prevent the spread of ‘toxins’ from an injured body part, and their descriptions suggest infection or gangrene. For example, one of Xue Ji’s cases described a man whose toes had turned black from some unnamed condition that had caused the ‘flesh to die’.^51^ Xue urged him to have the toes cut off, ‘otherwise, the dead flesh will spread into the foot, and we definitely will not be able to save [your life].’ The man refused, whereafter ‘the rot ascended into his shins and he died’. Hu Tingguang faced a similar problem in a case involving his younger brother’s wife. This sister-in-law was cutting open a fish, when a fishbone stabbed her under the nail of her index finger. They were unable to get the bone out, and over the next ten days, the wound worsened, with the fingertip becoming ‘suppurated and rotten’. When they finally consulted Hu Tingguang, he found that ‘the rot was about to reach the joint’ of her finger, and the flesh around the fishbone was black. ‘I immediately ordered that it [her fingertip] be cut off, along with the nail’, Hu recalled, and he explained that her life would be in danger otherwise.

If this affliction had been treated early, one could have avoided things reaching this stage. But since things have reached this stage, if one does not quickly cut it off, then the inevitable trend is for [the rot] to creep past the joint. As soon as it entered the palm of the hand, one would be unable to save her with medicine.^52^

Hu does not specify whether he personally performed the amputation, but the fingertip was indeed cut off. Afterwards, Hu washed the finger clean with a decoction of scallions (a standard treatment for wounds), filled an empty silkworm cocoon with wound medicine, and fitted it over her finger as a dressing. The finger subsequently healed.

## Bone Setting and Medical Skill

In cases of wounds to skin and flesh, the doctor could directly see and handle the injured parts. However, broken and dislocated bones were generally hidden from view. To properly diagnose and treat fractures, the doctor had to rely on his sense of touch. Citing the *Golden Mirror*, Hu’s section on manual orthopaedic methods started by discussing the technique of ‘feeling’ (*mo*):

‘Feeling’ means to use the hand to meticulously feel the injured place, [to determine] whether the bone is broken in two, or the bone is broken in pieces, or the bone is askew, or the bone is straight, or the bone is soft, or the bone is hard; or the sinew is tight, or the sinew is soft, or the sinew is askew, or the sinew is straight, or the sinew is broken in two, or the sinew has departed, or the sinew is thick, or the sinew is turned over, or the sinew is cold, or the sinew is hot.^53^

The high level of skill this required is shown in an official biographical account of Jueluo Yisang’a, a famous eighteenth-century Manchu bone setter. According to the *Draft History of the Qing* (*Qing shi gao*), ‘The way he taught his disciples was to cut up the shaft of a writing brush into many pieces, and wrap it in paper, and then have them palpate it manually and join up all the ends together, until it resembled its previously unbroken form. Then they would join bones in this matter, and in all cases it proved effective.’^54^

The Hu family cases show how these manual skills were applied in practice. When a farmer fell off his ox and landed on his knee, Hu’s father ‘palpated it with his hand and it made a rustling sound’, indicating a fracture. He treated the farmer by correcting the position of the knee cap and fixing it in place with a brace while it healed.^55^ When Hu Tingguang’s 12-year-old cousin tripped on a tree root and fell, hitting his arm, Hu ‘pressed on the spot’ and determined that it was ‘a slanted fracture’ and he set the bone accordingly.^56^ Another cousin was horsing around with a friend and fell, breaking two of his left ribs, confirmed by the fact that ‘the pointed ends of the ribs were protruding outwards’.^57^ Hu warned his readers that in cases of thin people one could readily discern a rib fracture ‘through touching’ but that if the person were fat, it might be difficult to get an accurate reading.

Proper diagnosis was important, of course, because the way that the healer treated the injury depended on the nature of the fracture. For example, fractured limbs were typically immobilised with strips of a stiff material (wood or bark) bound with lengths of cloth. For a ‘slanted fracture’ Hu taught, the binding should be tight in the middle and loose on the two ends, while for splintered (comminuted) fractures the binding should be loose in the middle and tight at the ends.^58^ A particularly difficult case involved a merchant who was struck in the lower leg during a group affray. Hu Tingguang said that he ‘palpated it above and below, and the fracture was like severed bamboo’. He then ‘gathered and aligned’ the pieces of bone in the merchant’s injured leg, and applied a plaster and a splint. But the injury was so serious that Hu ordered the merchant’s leg to be completely immobilised: ‘I used a cloth sock filled with grain to press upon [the leg] and hold it firm, and did not allow him to change position or move [the limb].’ The leg was inspected every five days, and the dressings changed every 10 days. Concurrently, the merchant was dosed with decoctions to regulate blood and promote bone healing. Before two months had elapsed, he was able to walk again.^59^

In the case of the arm-wrestling friends that opened this paper, Hu talked about ‘pressing’ the broken bone back into place. This reminds us that while bone setting required manual sensitivity, it also demanded physical exertion. This is especially clear in Hu’s cases of people with dislocated shoulders or hips. Anatomically speaking, both are major ball-and-socket joints held in place by a dense system of ligaments, tendons and muscles. To properly reduce a dislocation required the practitioner understanding the geometry of a given dislocation and to apply traction or leverage in the appropriate direction, while counteracting the resistance from traumatised muscles. Like their Western contemporaries, late imperial Chinese doctors recognised numerous methods for ‘putting up’ or ‘mounting’ (*shang*) a dislocated bone back to its original position. Each method had its advantages and disadvantages when judged by factors such as the number of people needed to perform the manoeuvre, the length of time it took, the level of pain inflicted on the patient and the general expectation of success. Hu used his medical cases to demonstrate the relative utility of different reduction methods, while also showing how one could improvise when standard methods were impracticable.

### Dislocated shoulders

One patient was a cart driver who dislocated his shoulder after falling off his ox. It is unclear whether Hu Tingguang was riding in the oxcart or just passing by, but when the driver asked him for help, there were only the two of them there. As Hu recalled, ‘I had no implements, and no one around to assist.’ This meant that well known methods for reducing shoulder dislocation could not be used. Hu talked about many of these methods in the section of his *Compilation* that described the structure of the ‘shoulder bone’ (*yu*) that served as a socket for the humerus (*nao*).^60^ One method, originally described by Wei Yilin in 1337, required considerable coordination.^61^ The patient stood on a low stool, while a long pestle was positioned with one end planted on the ground and the other lodged into the armpit of the affected shoulder. Then one person would hold fast the pestle, a second person would pull the patient’s afflicted arm outwards, and third person would stabilise the patient while he lowered himself into a sitting position. As the weight of the patient’s body sank onto the end of the pestle in his armpit, the bone would go back into place. If one did not have a stool, Wei advised that the same effect could be achieved by placing a wooden staff horizontally between the legs of two ladders. The injured person would position himself between the ladders, place this horizontal bar under his afflicted armpit, and again lower himself onto it. Hu Tingguang felt this method was quite time consuming, and he mentioned it only to say that there were better ways.

**Fig. 1:**
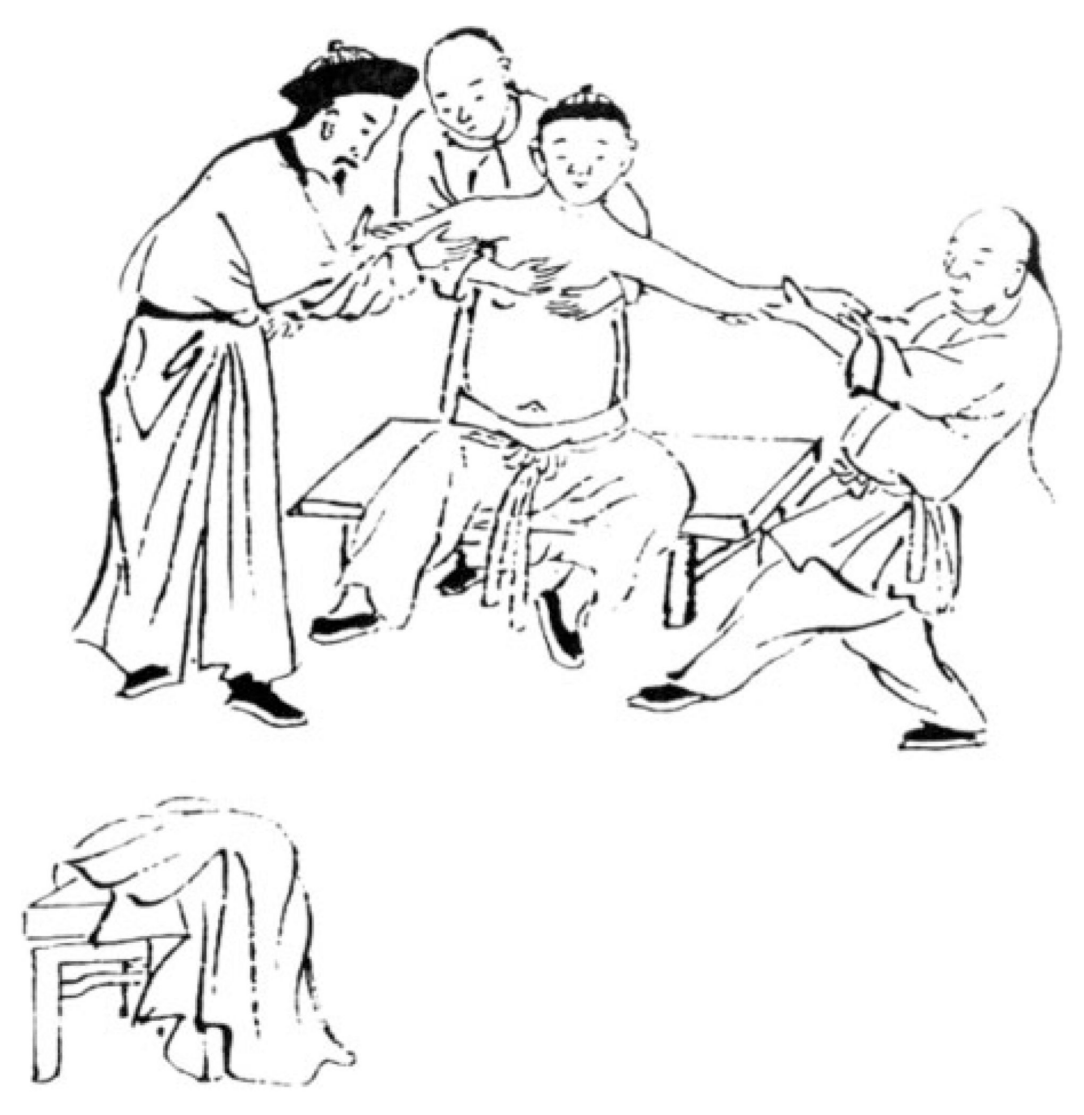
“Method for reducing a shoulder joint by manually pulling from both sides” (plate detail), Hu Tingguang, *Shangke hui zuan*, manuscript from the rare book collection of the Beijing University Library. Reproduced from the facsimile reprint published in Fu, et al., eds., *Beijing daxue tushuguan*, vol. 8, tu zhu (annotated images section): 17b. Digitally retouched for clarity by Yi-Li Wu

One method that Hu recommended, which he also depicted in an illustration, was adapted from the Monastery of the Lower Regions in Shanyin, a lineage of bone setting monks who claimed to have learned their techniques from the Shaolin Monastery. One person would hold the patient about the waist, bracing him, while two other people would each take hold of an arm and simultaneously pull outwards (Figure 1). But no such assistance was available when the cart driver fell off his ox. Hu therefore used a technique that he said was developed by his family: he inserted his own shoulder into the patient’s armpit and lifted him up. ‘As soon as I took him upon my shoulder, it went in,’ Hu said, ‘and his hand was able to raise and move.’ In his discussion of the shoulder bone, Hu explained that this technique worked because, ‘The afflicted person’s body is heavy and hangs downwards, and moreover the afflicted arm is being held firmly downwards by the doctor with both hands. The force is not insignificant, and the humerus will enter the socket, and the seam will close.’^62^ While it is beyond the scope of this paper to detail all the other methods that Hu discussed, it is worth noting that similar techniques were historically used in societies around the world. The method of taking the patient upon the healer’s shoulder was also described in the Hippocratic corpus, and Chinese methods of rotating and lifting the injured arm would have been familiar to German doctors of the nineteenth century ce as well as to Egyptian doctors of the thirteenth century bce.^63^

### Dislocated hips

Chinese doctors viewed hip dislocation as a serious condition that could require forceful manipulations to resolve. For example, Master Chen’s bone setting book felt that a hip dislocation was ‘the most difficult illness condition (*zheng*) to treat’.^64^ In his discussion of the hip bones (*kua gu*) and sockets (*huan tiao gu*), Hu Tingguang elaborated on three methods of reduction. The first required one person to hold the patient and another to pull on his injured leg. The second, which relied on suspending the person upside down, was also elaborated in Hu’s case of a sailor who dislocated his hip after falling into the hold of a grain boat. Hu bound the sailor’s lower legs to the mast ropes and had someone hoist him up. While the sailor was thus suspended, Hu used his hands to press the bone back into the joint.^65^

The third technique, in which the practitioner used his own body as a traction device, was something Hu suggests he invented himself.^66^ One day, Hu was out walking in the countryside when he came across a young man who had fallen off his horse, dislocating his hip. No one else was around to help. So Hu got down on the ground and planted his foot into the buttock of the young man’s affected hip, while grasping the shin of that leg with both hands. He then pulled back on the leg while simultaneously pushing upwards into the buttock with his foot. Once the bone went back in, the man got up, ‘made a bow of thanks’, and slowly walked off to try to find his horse. This marvelous cure, Hu said, showed that one needed to be flexible when treating injuries. ‘If I had been mired in the old ways of asking many people to help,’ Hu pointed out, ‘then not only would he have had to pass through many more difficulties, but I fear that the delay would have led to things becoming grave. Then even if one wanted him to rise up and stand in just a moment, how would that be possible?’ Thereafter, Hu said, he often used this method with great success. However, he warned, one could not use it on women because it violated the norms of propriety. Hu thus devoted several cases to teaching his readers how to reduce dislocations in women without handling their bodies. Here virtuosity lay in knowing how to deploy both psychological and physical manipulations.

### Gender norms and traumatology

Late imperial Chinese gender norms forbade physical contact between unrelated women and men. This meant, among other things, that midwives and other female practitioners were an important source of medical care for women.^67^ In cases of illness or injury, however, gender segregation was negotiable. Medical case histories show that male doctors were in fact able to treat women for a wide range of ailments, including maladies related to menstruation, pregnancy and childbirth.^68^ Doctors’ cases also routinely report women’s pulse signs, suggesting there was no significant impediment to men palpating the female wrist. Overall, in cases where the therapies consisted of drugs that the woman or her family could administer themselves, gender norms did not necessarily prevent male doctors from treating female patients.

Hu’s cases show, however, that the negotiation of gender norms was more complex when the doctor needed to grasp and manipulate the body. Body parts could differ as to their erotic or gendered meanings. It appears that touching a woman’s head was not particularly problematic, and boundaries also seemed more relaxed for children and women past childbearing age. We saw above that Hu Tingguang treated a girl for a bleeding wound by binding a felt cap to her head.^69^ Likewise, his father cured a girl of seven who had fallen out of a window, causing her neck bones to be ‘retracted into her body’. Hu’s father applied upwards traction on the girl’s head by cupping his hand under her chin and pulling up on her hair.^70^ Both father and son treated the 60-year-old woman whose jaw repeatedly dislocated. These case narratives contain no hint of gendered anxieties.

With other body parts, however, gender propriety could become an issue, especially if the patient was a young woman. One of Hu Tingguang’s patients was a married woman of 19 who wrenched her ankle after slipping on muddy ground. ‘Her right ankle bone was sticking out on the exterior,’ Hu recalled, and he would have to hold her lower leg—including her bound foot—to manipulate the bone back into place. Although early Western accounts emphasised the sexual allure attributed to a woman’s bound foot, Dorothy Ko has shown that the bound foot was fundamentally a symbol of a woman’s chastity, propriety and diligence.^71^ A proper woman would not normally show her foot to an unrelated man, let alone allow him to handle it. Hu thus wondered how he could find ‘something to hold onto’ in order to reduce the dislocation. He solved the problem by having the woman put her husband’s sock on over the injured foot. With the spousal garment serving as an implied shield against the touch of the male doctor, Hu was able to ‘rub’ the young wife’s dislocated ankle back into place.^72^

By comparison, cases of dislocated shoulders or hips normally required the doctor to view and touch the patient’s torso, especially the armpit and groin area. Gender propriety could very well prohibit the doctor from carrying out the usual procedures on female patients. Hu Tingguang therefore described various remote traction and psychological manipulation techniques that one could employ in such instances.^73^ These techniques were not unique to the Hu family, for Hu Tingguang mentioned that they were used by the Monastery of the Lower Regions in Shanyin. But in his family’s medical cases, Hu gave examples of how these techniques were used in practice while also attesting to their efficacy.

### Remote traction and scare tactics

**Fig. 2:**
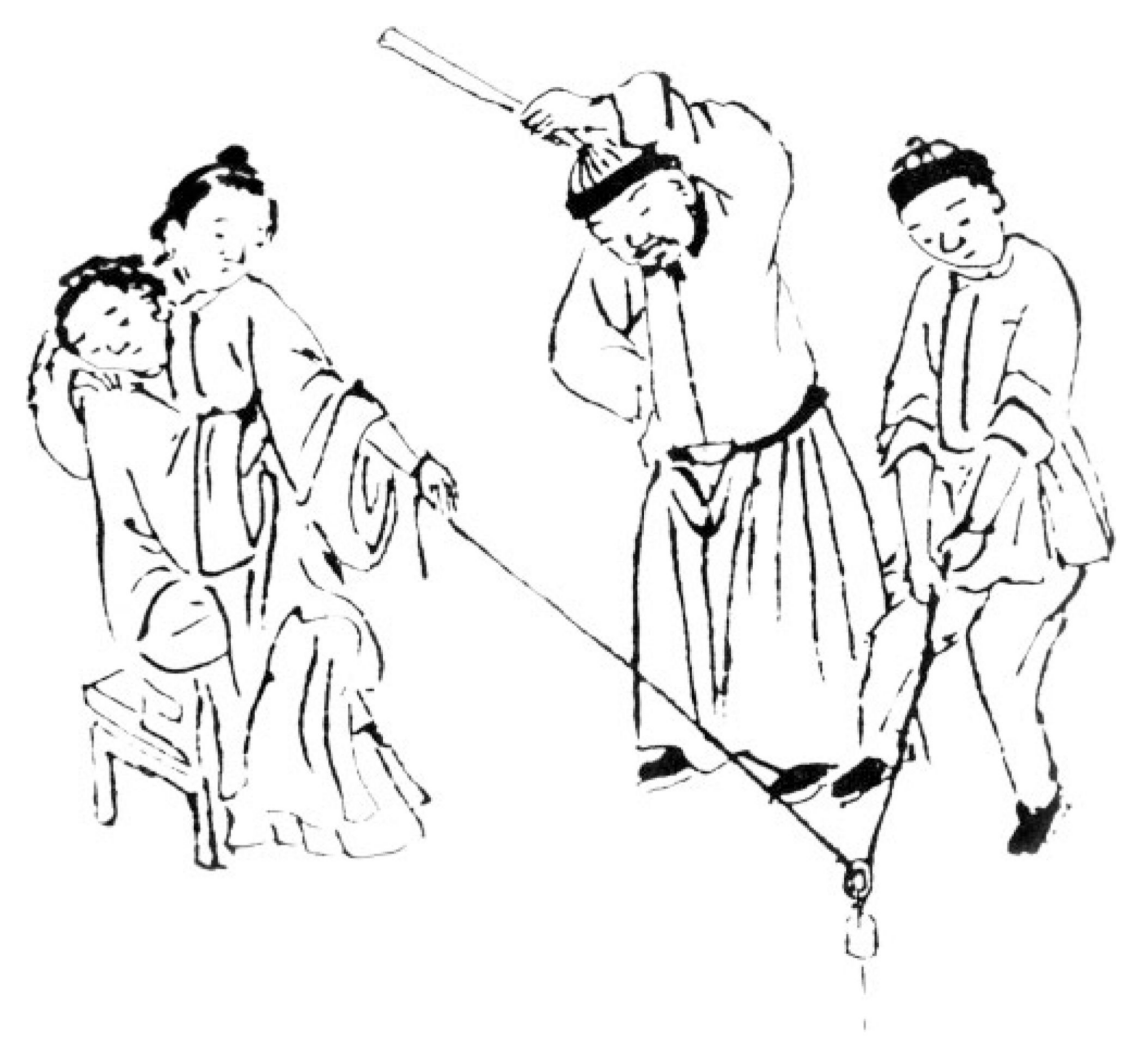
“Method for mounting a shoulder joint using a belt that is attached, fixed, and hit” (plate detail), Hu Tingguang, *Shangke hui zuan*, manuscript from the rare book collection of the Beijing University Library. Reproduced from the facsimile reprint published in Fu, et al., eds., *Beijing daxue tushuguan*, vol. 8, tu zhu:18b. Digitally retouched for clarity by Yi-Li Wu

What I call ‘remote traction techniques’ were those in which the doctor tied a rope or cloth band to the afflicted limb, then pulled on it from a remove. To preserve the woman’s modesty, the doctor might even go to an adjoining room and pull on the limb through a window or doorway. One of Hu’s illustrations depicts a remote traction technique for reducing a shoulder dislocation by ‘using a belt that is attached, fixed and hit’ (Figure 2). It shows a cord tied to the woman’s wrist, running downwards through an eyelet-like object that appears tethered to a fixed point on the ground. One man is pulling upwards on the free end of the cord, while another is using a stick to strike the portion of the cord near the arm. The accompanying caption explains that after lulling the woman into inattention by repeatedly hitting the tensed cord, one should suddenly pull hard on it to make the bone go back in. Hu Tingguang explained how he used this technique to treat a young married woman with a posterior dislocation of the shoulder:

Because she was young and delicate and bashful, it was difficult to carry out manual methods. So I ordered her mother to tightly clasp her while sitting on a chair. I used a cloth to make a connecting strip, with one end tied firmly to her hand, and one end threaded underneath the door sill. From the adjoining room I pulled it taut. I also used a wooden ruler to strike the connecting strip, as if teasing out cotton with a bow. Then I waited until her guard was down, and suddenly pulled hard, and the bone went into the socket.^74^

Hu also taught his readers how to treat women with ‘scare tactics’ (*xia fa*) and ‘deception’ (*hong pian*) techniques. The principle was to elicit a sudden, involuntary reaction that would make the woman move her afflicted limbs in a therapeutically productive way. The medical utility of deception and fright was already well-attested in works such as Wu Kun, *Investigations into Medical Remedies* (*Yi fang kao*, 1584). Wu’s section on illnesses caused by excessive emotion related several cases where doctors cured the patient by provoking another strong emotion that, according to Five Phases cosmological thinking, could counteract the original emotion and thereby harmonise bodily vitalities.^75^ To provoke the desired emotion, the doctor might tell the patient a lie or act in a threatening manner. The Hu family cases show that such psychological manipulations could also be used to address injuries to the body’s structures. One patient thus cured by Hu Tingguang’s father was a young married woman whose arms became stuck above her head after she strained to reach a high object.^76^ She refused to allow Hu’s father to touch her shoulders. So he instructed her family to tie her to a pillar in the courtyard, and to tell her (falsely) that the doctor was going to cure her by needling her leg. As they removed her outer skirt, she protested that she preferred to remain a cripple. As they started to remove her inner skirt, she began screaming in rage. In desperation, ‘she frantically assumed the position of one facing an oncoming enemy, and her two hands came down together’. This panic reaction cured her shoulder affliction.

**Fig. 3:**
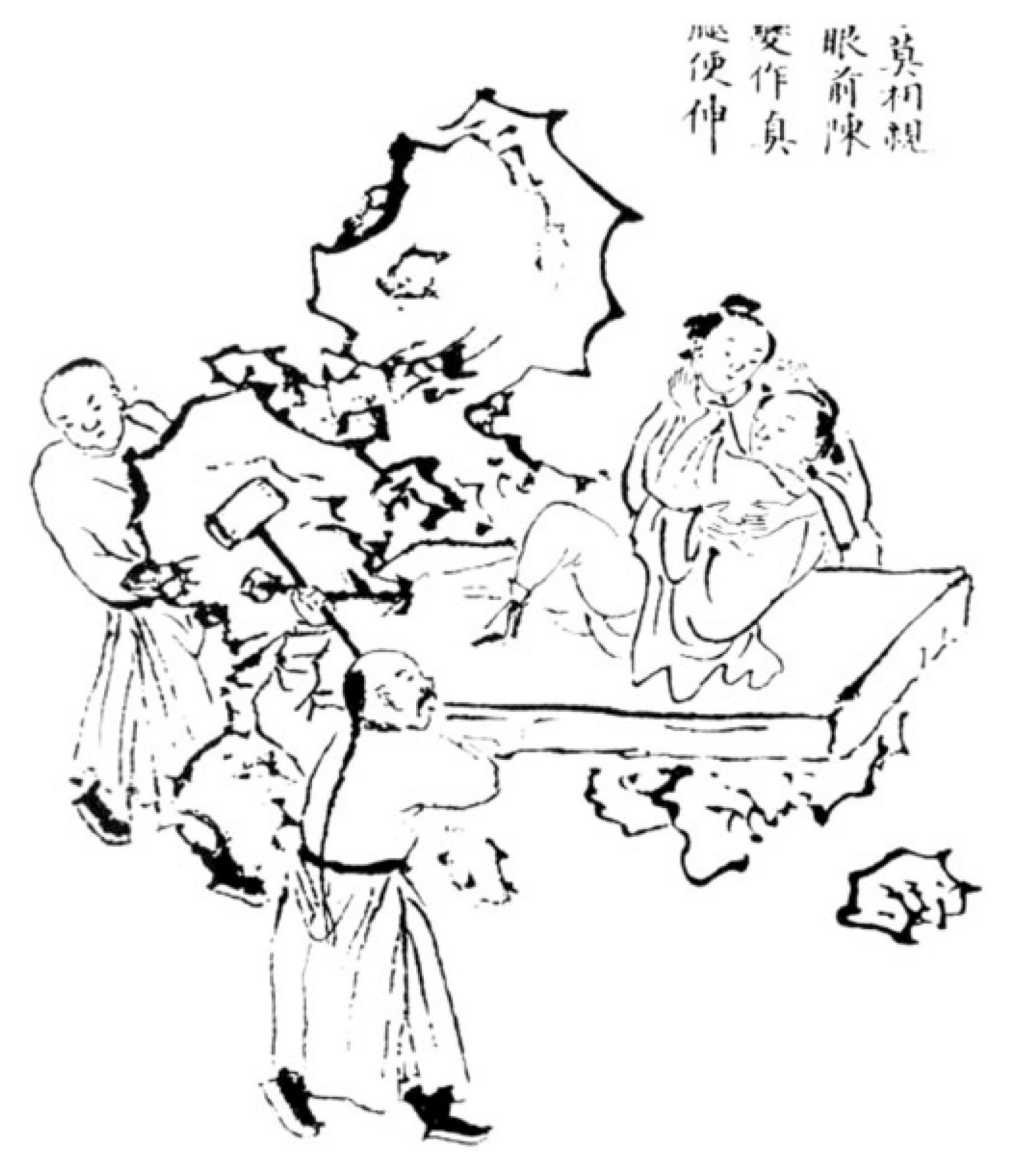
“Method for mounting a hip joint using the mallet scare method” (plate detail), Hu Tingguang, *Shangke hui zuan*, manuscript from the rare book collection of the Beijing University Library. Reproduced from the facsimile reprint published in Fu, et al., eds., *Beijing daxue tushuguan*, vol. 8, tu zhu:22a. Digitally retouched for clarity by Yi-Li Wu

Scare techniques could also be used in conjunction with remote traction. While one person pulled on the afflicted limb, the other would frighten the woman into jerking her body, thereby generating a combined force that would pull the bone back to its original position. One of Hu’s diagrams showed how to reduce a hip dislocation with the ‘mallet scare method’ (Figure 3). While traction was applied to the patient’s dislocated leg, the doctor would pretend that he was going to hit her leg with a heavy mallet, thus causing her to contract in fright. Hu explains how his father used this method to treat a woman who had fallen while putting up frames for raising silkworms, sustaining a posterior dislocation of the hip:

Although she was a peasant woman, using a hand to press on her lower body was still indecent. My late father set up two large mallets, one solid and one hollow. The solid one was made of sandalwood and weighed over 30 *jin*. The empty one was made of cowskin, and was light, only one or two *jin*. He first took the heavy one and put it in front of the afflicted person, and let it make a clanging noise. Then he had the afflicted woman lie sideways on the ground, with her afflicted leg on top. A woman pressed and held firm her body. Moreover, they took the foot of the injured leg and firmly tied a girdle sash to it. He placed himself in the next room and pulled it taut. Secretly he had someone take the heavy mallet and replace it with the light one. They lifted it high, aiming at the dislocation, and hit her to frighten her. The afflicted woman was alarmed and timid, and her sinews and bones contracted tightly, and without her feeling any pain, the leg bone went back into the socket.^77^

These were not simple methods to employ, and Hu Tingguang cautioned that one had to tailor the techniques to the situation at hand. Judging from the family cases, Hu Tingguang’s father was especially skilled at reading his patients and understanding what psychological strategies might be used. One troublesome case involved the beautiful teenage concubine of an official.^78^ She had fallen down the stairs, dislocating her leg ‘to the exterior’. The medical case narrative described the concubine’s lightweight summer clothing in detail, hinting at the erotic tensions that could arise in such encounters: ‘Her inner garment was of blood red light silk gauze, through which one could faintly see her body. On the outside, she wore a cicada coloured skirt of thin silk. She fluttered like a divinity, and truly was like a being from heaven.’ Hu’s father initially attempted to reduce the dislocation by remote traction, calculating that ‘the method of frightening her would be difficult to carry out’. So a serving woman held the concubine’s body firm, while Hu’s father went into the next room and pulled on a cloth sash tied to the injured leg. However, ‘the beauty’s cries [of pain] were frightful, and he concluded that there would be no outcome’. At this point, Hu’s father went back into the concubine’s room, carrying a fan in his hand. He deliberately fanned a gust in her direction, causing her diaphanous clothing to blow up and completely expose her bare skin. This did the trick: ‘The concubine was bashful and quickly drew back, and without her noticing, the leg bone went back in the joint by itself.’

## Conclusion

Historians of Chinese medicine must contend with a basic source bias: the healers who produced the most written records—the literate male doctors—played only a minor role in providing health care to ordinary people. As Nathan Sivin emphasises, the vast majority of healing services came from folk healers—including midwives, herbalists, bone setters and ritual experts—possessing varying levels of literacy, who typically learned their skills from family members or masters.^79^ In his study of rural health care in Maoist China, Xiaoping Fang describes the life of Shen Jinrong, a folk healer who was recruited into the ranks of the ‘barefoot doctors’. Jinrong had originally learned healing from his grandfather Shen Fengxiang (1861–1940), who was an expert in bone setting, the treatment of injuries and bloodletting (a popular therapy for ‘heat stroke’).^80^ Hu Tingguang might also be labelled a folk healer by virtue of the kind of healing that he practised: sewing people up, joining their bones, cutting their flesh. As a literate man, however, Hu sought to integrate these manual techniques into a larger discourse of scholarly medicine, turning oral lore into textual teachings, and using his own experiences and perspectives to provide a model of insightful judgment and therapeutic virtuosity. Hu’s cases thus provide a valuable window onto the kind of therapies that healers like the Shens might have used. By showing the level of expertise to which healers might aspire, Hu also helps us understand the range of healing resources available to ordinary Chinese of the past.

But beyond simply filling in historical lacunae, Hu’s *Compilation* invites us to reconsider the place of manual healing in Chinese medicine and how we think about its historical development. The dominant historiographical narrative is that after the Song dynasty (960–1279), manual healing—including surgery—was ‘marginalised’ or ‘declined’ in China as scholarly modes of medical practice began to expand. As one authoritative account notes, ‘…erudite physicians tended to relegate hands-on practices such as acupuncture, bone setting, minor surgeries, the treatment of skin diseases, massage, and ritual therapies to less prestigious practitioners denigrated … as technical specialists’.^81^ A leading historian of surgery in China has also argued that manual techniques became neglected as a result of the ‘internal medicine-ization’ (*neike hua*) of ‘external ailments’ (*waike*): learned medicine increasingly redefined skin and flesh ailments as the product of internal imbalances, to be treated with oral decoctions rather than petty surgery.^82^

But while these narratives do describe important trends in Chinese medical history, the story of decline does not provide satisfactory context for the descriptions of petty surgery that continue to appear in post-Song textual records. Even Xue Ji, whose doctrinal innovations epitomised the ‘internal medicine-ization’ of external ailments, routinely wielded the lancet, and he argued that failure to cut away diseased flesh when necessary would lead to the patient’s death.^83^ Similarly, Hu Tingguang’s *Compilation* both supports and challenges the narrative of decline and marginalisation. Hu himself complained that there was a lack of specialised writing on traumatology, and that educated doctors were insufficiently schooled in these practices. At the same time, however, his textual project shows that traumatology and petty surgery were active arenas of practice and knowledge formation, even if this knowledge only sporadically appeared in printed texts. Many of the *Golden Mirror*’s descriptions of bone setting are entirely new to the medical literature, for example. These were most likely based on the techniques of Mongol bone setters, who were famed for their prowess in treating injuries but assigned to the government’s equestrian administration, rather than the Imperial Medical Academy.^84^ When Hu Tingguang expanded the imperial text, he likewise wrote down techniques that had previously been transmitted only orally.

More research is needed to understand the world of healing that lay outside texts, as well as the circumstances in which such knowledge entered the textual sphere. Hu’s ambitious *Compilation*, meanwhile, underscores the fact that medical change is not unidirectional, and that apparent signs of decline can coexist with episodes of innovation. Indeed, Hu’s effort to create a comprehensive ‘curriculum of injuries’ was itself an innovation. Furthermore, as mentioned briefly above, Hu was not the only one thinking along these lines, as his contemporary Qian Xiuchang also wrote a work on traumatology meant to improve on the *Golden Mirror*. One might even hypothesise that the late eighteenth and early nineteenth centuries witnessed a growing convergence between the realm of traumatology and the interests of literate men. Notably, it was during this same period that Chinese forensic practice paid increasing attention to the problem of wounds, drawing on medical knowledge about injuries to adjudicate cases of assault and battery.^85^ In sum, while Hu Tingguang’s *Compilation* is extraordinary in many ways, it was also a product of its time. It thus demonstrates the necessity for and value of additional research into the history of the structural body and manual therapies in China.

## Footnotes

1 Hu Tingguang, author’s preface dated 1815, *Shangke hui zuan* (A Compilation of Teachings on Traumatology), manuscript held by the Beijing University Library, facsimile reprint published in *Xu xiu Si ku quan shu* (A continuation of the *Imperial Library of the Four Treasuries*) (Shanghai: Shanghai guji chubanshe, 1995–2002), 6:47b. Unless otherwise noted, all citations refer to this edition. My figures come from the slightly clearer facsimile of the Beijing manuscript published in Fu Jinghua, Xu Yanchun, Du Jiuhui, eds, *Beijing daxue tushu guan guan cang shanben yishu* (Rare Medical Books Held by the Beijing University Library), (Beijing: Zhongyi guji chubanshe, 1988), Second series, vols 8–9. I have also consulted a facsimile reprint of the manuscript held by the Sun Yat-Sen University Library in Guangzhou, published in Sang Bing, ed., *Si bian Qingdai gao chao ben* (A Fourth Compilation of Qing Dynasty Manuscripts and Handcopied Texts) (Guangzhou: Guangdong renmin chubanshe, 2012), vol. 188.

2 Carol Benedict, *Bubonic Plague in Nineteenth-Century China* (Stanford: Stanford University Press, 1996); Marta E. Hanson, *Speaking of Epidemics in Chinese Medicine: Disease and the Geographic Imagination in Late Imperial China* (Abingdon: Routledge, 2011); Angela Ki Che Leung, *Leprosy in China: A History* (New York: Columbia University Press, 2009); Chang Chia-feng, ‘Aspects of Smallpox and its Significance in Chinese History’ (unpublished PhD diss, University of London, 1996); Fabien Simonis, Mad Acts, Mad Speech, and Mad People in Late Imperial Chinese Law and Medicine (unpublished PhD diss., Princeton University, 2010); Hilary A. Smith, ‘Foot qi: History of a Chinese Medical Disorder’ (unpublished PhD diss., University of Pennsylvania, 2008).

3 Li Jianmin, *Hua Tuo yincang de shoushu: Waike de zhongguo yixue shi* (The Secretly Kept Manual Techniques of Hua Tuo: A Chinese Medical History of Surgery) (Taipei: Dongda tushu gongsi, 2011); Jürgen Kovacs and Paul U. Unschuld, transl. and annot., *Essential Subtleties on the Silver Sea: The Yin-hai jing-wei: A Chinese Classic on Ophthalmology* (Berkeley: University of California Press, 1998); Charlotte Furth, *A Flourishing Yin: Gender in China’s Medical History, 960–1665* (Berkeley: University of California Press, 1999); Yi-Li Wu, *Reproducing Women: Metaphor, Medicine, and Childbirth in Late Imperial China* (Berkeley: University of California Press, 2010); Hsiung Ping-chen, *Youyou: Chuantong zhongguo de qiangbao zhi dao* (To Care for the Young: The Way of the Swaddling Cloth in Traditional China) (Taipei: Lianjing, 1995).

4 See notably Ding Jihua, ed., *Shangke ji cheng* (Compendium of Medicine for Injuries) (Beijing: Renmin weisheng chubanshe, 1999).

5 See, for example, Mei Zhan, *Other-Worldly: Making Chinese Medicine Through Transnational Frames* (Durham, NC: Duke University Press, 2009).

6 Bridie Andrews, *The Making of Modern Chinese Medicine, 1850–1960* (Vancouver, BC: UBC Press, 2014); Sean Hsiang-lin Lei, *Neither Donkey nor Horse: Medicine in the Struggle over China’s Modernity* (Chicago: University of Chicago Press, 2014); Volker Scheid, *Currents of Tradition in Chinese Medicine: 1626–2006* (Seattle: Eastland Press, 2007); Kim Taylor, *Chinese Medicine in Early Communist China, 1945–1963: A Medicine of Revolution* (London: RoutledgeCurzon, 2005).

7 Lei, *Neither Donkey nor Horse*.

8 Yi-Li Wu, ‘Bodily Knowledge and Western Learning in Late Imperial China: The Case of Wang Shixiong (1808–68)’, in Howard Chiang, ed., *Historical Epistemology and the Making of Modern Chinese Medicine* (Manchester: University of Manchester Press, 2015), 80–112.

9 Eric Karchmer, ‘Slow Medicine: How Chinese Medicine Became Efficacious only for Chronic Conditions’, in Chiang, ed., *Historical Epistemology*, 188–216.

10 Hu, *Shangke*, *fanli* (‘Guide for the reader’).

11 See Wu, *Reproducing Women*, ch. 1.

12 Hu, *Shangke*, *fanli*.

13 *Ibid*.

14 For example, in the official histories compiled by counties and prefectures, the biographies of doctors were classified under ‘experts in methods and techniques’ (*fang ji*).

15 For cases involving upper class clients, see Hu, *Shangke*, 6:46a, 6:54b, 6:55b, 6:58a.

16 James Cole, *Shaohsing: Competition and Cooperation in Nineteenth-Century China* (Tucson: The University of Arizona Press, 1986).

17 Hu, *Shangke*, 6:58a–b. Two other descriptions that suggest Hu Tingguang went to Beijing are found at 6:46a and 6:55a.

18 *Ibid*., prefaces by Sheng Tang and Yu Dengyuan.

19 *Ibid*., author’s preface.

20 See Xue Qinglu, ed., *Zhongguo zhongyi guji zongmu* (General Catalog of Ancient Medical Books on Chinese Medicine) (Shanghai: Shanghai cishu chubanshe, 2007).

21 The most important difference is that the Guangzhou version does not include Hu’s medical cases. I suspect it is an earlier version, and that the medical cases were added when the Beijing copy was produced.

22 For medical specialisation, see Wu, *Reproducing Women*, ch. 1.

23 Hu, *Shangke*, author’s preface.

24 Marta E. Hanson, ‘The Golden Mirror in the Imperial Court of the Qianlong Emperor’, *Early Science and Medicine: Special Issue: Science and State Patronage in Early Modern East Asia*, 8, 2003, 111–47.

25 Yi-Li Wu, ‘Between the Living and the Dead: Traumatic Injuries and Forensic Medicine in the mid-Qing’, *Frontiers of History in China*, 10, 2015, 38–73.

26 For the Korean doctors see Hu, *Shangke*, 5:15b, For Adam Schall von Bell, see *ibid*., 2:4a–b.

27 Chen Chong-Fang, ‘“Qing “Lüliguan jiaozheng xiyuan lu’ xiangguan wenti kaozheng (A textual study of questions pertaining to the Qing *Records on the Washing Away of Wrongs, edited by the Codification Office*)”’, *Youfeng chuming niankan*, 6, 2010, 441–55.

28 For forensic-medical overlaps, see Wu, ‘Between the Living and the Dead’.

29 Hu, *Shangke*, *fanli*.

30 For the similarities between Qian’s and Hu’s texts, see Wu, ‘Between the Living and the Dead’.

31 For aetiology in scholarly medicine see Hanson, *Speaking of Epidemics*.

32 Hu, *Shangke*, 5:26a–29a, 6:7a–10a.

33 Compare, for example, Hu’s discussion of injuries caused by metal implements, bricks and stones (Hu, *Shangke*, 9:30a, 9:32a) with *Lüliguan jiaozheng xi yuan lu* (*Records on the Washing Away of Wrongs*, edited by the Codification Office) (eighteenth century; reproduced in the *Xu xiu Si ku quan shu*), 2:5a–b.

34 Hu, *Shangke*, ‘Shangke bencao zhuzhi,’ [section following *juan* 8], s.p. 1a.

35 For medical amateurs, see Wu, *Reproducing Women*, ch. 2.

36 Hu, *Shangke*, author’s preface.

37 Xue Ji, *Zhengti lei yao* (Classified Essentials of Rectifying the Body) (*c*. 1548, edition in *Xue shi yi’an* [Master Xue’s Medical Cases], reprinted in the *Si ku quan shu*), Lu Shidao preface.

38 Hu, *Shangke*, 6:44a.

39 Charlotte Furth, Judith T. Zeitlin and Ping-chen Hsiung, eds, *Thinking with Cases: Specialist Knowledge in Chinese Cultural History* (Honolulu: University of Hawai‘i Press, 2007); Christopher Cullen, ‘*Yi’an* (Case Statements): The Origins of a Genre of Chinese Medical Literature’, in *Innovation in Chinese Medicine*, ed. Elisabeth Hsu (Cambridge: Cambridge University Press, 2001), 297–323; and Joanna Grant, *A Chinese Physician: Wang Ji and the ‘Stone Mountain medical case histories’* (London and New York: RoutledgeCurzon, 2003).

40 Hu, *Shangke*, 6:44a.

41 *Ibid.*, 6:44b.

42 *Ibid.*, 6:45b.

43 *Ibid.*, 6:48b–49b.

44 *Ibid.*, 6:49b.

45 Hu, *Shangke*, 6:50b.

46 *Ibid.,* 6:52a–b. For early discussions of surgical treatment, see Chao Yuanfang, *Chaoshi zhubing yuanhou lun* (Master Chao’s treatise on the origins and manifestations of the myriad diseases) (compiled 610; Ming edition reprinted in the *Si ku quan shu* [Complete Libraries of the Four Treasuries], Wenyuan ge edition, 1782; facsimile reprint published Taipei: Taiwan shangwu yinshuguan, 1986), 36:10b, and Wei Yilin, *Shiyi de xiao fang* (Efficacious Prescriptions from a Hereditary Medical Family) (1337, reprinted in the *Si ku quan shu*), 18:6a–b. The rocking method appears in *Liu Juanzi gui yi fang* (Liu Juanzi’s Remedies Bequeathed by a Ghost), conventionally dated 499. See Ding, *Shangke*, 593.

47 Hu, *Shangke*, 53:a–b.

48 I have been unable to identify Master Jinxi. Hu’s account of him cites Chen Wenzhi, *Yang ke xuan cui* (Refined Selections on the Curriculum of Lesions, 1628). Hu, *Shangke* 6:11a. For the wall coin spider, see Li Shizhen, *Ben cao gang mu* (Systematic Outline of Materia Medica) (pref. dated 1590; modern reprint Zhongguo shudian 1988), 21, 101–2.

49 Hu, *Shangke,* 6:52b–53a.

50 See, for example, Wu Qian, ed., *Yu zuan yi zong jin jian* (The Imperially-Compiled Golden Mirror of Medical Learning) (1742; reprinted in the *Si ku quan shu*), 69:47b (haemorrhoids) and 73:46b (warts).

51 Xue, *Zhengti*, 11:35b–36a.

52 Hu, *Shangke*, 6:50a–b.

53 *Ibid.*, 3:2a

54 *Qing shi gao* (Draft History of the Qing Dynasty) (1927, reprinted Beijing: Zhonghua shuju, 1977), 502, 13880–81.

55 Hu, *Shangke*, 6:57b

56 *Ibid.*, 6:48b.

57 *Ibid.*, 6:51b.

58 *Ibid.*, 3:15b.

59 *Ibid.*, 6:57b–58a.

60 *Ibid.*, 6:18a–22b.

61 Wei, *Shiyi*, 11:2a–b.

62 Hu, *Shangke*, 6:21b.

63 See, for example, A. Mattick and J. P. Wyatt, ‘From Hippocrates to the Eskimo: A History of Techniques Used to Reduce Anterior Dislocation of the Shoulder’, *Journal of the Royal College of Surgeons of Edinburgh*, 45, 2000, 312–16 and M. Kamel Hussein, ‘Kocher’s Method is 3,000 Years Old’, *The Journal of Bone and Joint Surgery*, 50, 1968, 669–71.

64 Hu, *Shangke*, 6:32b.

65 *Ibid*., 6:55a–b.

66 Hu’s discussion of the hip joint describes the technique as something ‘transmitted in my family’. The narrative structure of his medical case, however, suggests that Hu himself invented it. Compare Hu, *Shangke*, 6:33b–34a and 6:56a–b.

67 Furth, *A Flourishing Yin*, ch. 8.

68 Furth, *A Flourishing Yin* and Wu, *Reproducing Women*.

69 Hu, *Shangke*, 6:44b–45a.

70 *Ibid.,* 6:45b–a.

71 Dorothy Ko, *Cinderella’s Sisters: A Revisionist History of Footbinding* (Berkeley: University of California Press, 2007).

72 Hu, *Shangke*, 6:58b–a.

73 Besides the cases discussed here, see also Hu, *Shangke*, 3:13a, 3:16a.

74 *Ibid.*, 6:46b.

75 See discussion in Nathan Sivin, ‘Emotional Counter-Therapy’, in Sivin, *Medicine, Philosophy and Religions in Ancient China: Researches and Reflections* (Aldershot: Variorum, 1995), 1–19 (s.p.).

76 Hu, *Shangke*, 6:47a–b.

77 *Ibid.*, 6:56b–57a.

78 *Ibid.*, 6:55b–56a.

79 Nathan Sivin, *Health Care in Eleventh-Century China* (New York: Springer, 2015).

80 Xiaoping Fang, *Barefoot Doctors and Western Medicine in China* (Rochester, NY: University of Rochester Press, 2012), 21–2, 36, 57.

81 T. J. Hinrichs and Linda Barnes, eds, *Chinese Medicine and Healing: An Illustrated History*. (Cambridge, MA: The Belknap Press of Harvard University Press, 2013), 116.

82 Li, *Hua Tuo*.

83 See his discussion of ‘the error of not using the lancet’, Xue, *Zheng ti lei yao*, 11:19b–20a.

84 See Sare Aricanli, ‘Plurality in Qing Imperial Medicine: Examining Institutional Formations Beyond the Imperial Medical Bureau’, *Asia Pacific Perspectives*, XII, 2014, 61–83 and Wu, ‘Between the Living and the Dead’.

85 Wu, ‘Between the Living and the Dead’.

